# Towards understanding foot mobilisation techniques: a pilot study evaluating the immediate effects

**DOI:** 10.1186/1757-1146-6-S1-O36

**Published:** 2013-05-31

**Authors:** Aaron Wholohan, Lloyd Reed, Scott Wearing, Sheree Nix, Ted Jedynak

**Affiliations:** 1 School of Clinical Sciences, Faculty of Health, Queensland University of Technology, Brisbane, Queensland, 4059, Australia

## Background

While considerable research has evaluated the effects of joint mobilisation on spinal, pelvic and shoulder biomechanics, there is a paucity of research evaluating the effects of foot mobilisation techniques (FMT) on gait. This pilot study evaluated the immediate effects of FMT on plantar pressures and temporal-spatial gait parameters.

## Methods

Fifteen adults (48±21.4 years) that had no known balance problems or falls history participated. An instrumented treadmill system (Zebris FMD-T, Zebris Medical, Germany) was used to measure plantar pressure and temporal-spatial gait parameters. Data were recorded for 30 seconds of steady state walking immediately before and after intervention using a standardised protocol of FMT. Repeated measures ANOVAs were used to assess the effect of FMT on gait parameters at an alpha level of .05.

## Results

Of the 34 gait parameters measured, only three changed significantly after FMT. Peak pressure beneath the lateral heel (4%) and lateral forefoot (9%) was increased immediately following FMT (p<.05) and was accompanied by a delay (3%) in the time to peak pressure beneath the lateral forefoot (p<.05).

## Conclusion

Changes in plantar pressure following FMT were small and less than the reported measurement error of the treadmill system. Therefore, in this pilot study the immediate effect of FMT on gait parameters was negligible. Further research evaluating short and long term effects of FMT on specific aspects of the locomotor system are needed.

